# Frontiers in Bladder Cancer Genomic Research

**DOI:** 10.3389/fonc.2021.670729

**Published:** 2021-05-20

**Authors:** Yi Li, Lihui Sun, Xiangyang Guo, Na Mo, Jinku Zhang, Chong Li

**Affiliations:** ^1^ Department of Anesthesiology, Peking University Third Hospital, Beijing, China; ^2^ Core Facility for Protein Research, Institute of Biophysics, Chinese Academy of Sciences, Beijing, China; ^3^ Department of Pathology, Beijing Obstetrics and Gynecology Hospital, Capital Medical University, Beijing, China; ^4^ Department of Pathology, First Central Hospital of Baoding, Baoding, China; ^5^ Key Laboratory of Molecular Pathology and Early Diagnosis of Tumor in Hebei Province, First Central Hospital of Baoding, Baoding, China; ^6^ Department of Immunology, Beijing Jianlan Institute of Medicine, Beijing, China; ^7^ Department of Immunology, Beijing Zhongke Jianlan Biotechnology Co., Ltd., Beijing, China

**Keywords:** bladder cancer genomics, heterogeneity, epigenetics, oncogene sequencing method, precision treatment

## Abstract

Most of the etiology studies of bladder cancer focus on genetic changes, mainly including mutation and activation of oncogenes, mutation and inactivation of tumor suppressor genes, and rearrangement or heterozygous deletion of chromosomes. Moreover, bladder cancer is highly heterogeneous mainly due to abnormal changes in the genome and proteome of tumor cells. Surgery is the main treatment for bladder cancer, but because the recurrence rate is high after surgery and most of the muscle-invasive bladder cancer acquires distant metastasis. Therefore, there is a need to combine with chemotherapy to consolidate the treatment effect. However, there are differences in chemosensitivity among patients. In this article, we review the up-to-date genomic researches on bladder cancer occurrence, development, metastasis, and chemosensitivity in patients, in order to provide some theoretical support for the diagnosis and treatment strategy for bladder cancer.

## Introduction

Bladder cancer is the ninth most common malignant disease worldwide, with 549,393 new cases reported in 2018, and it ranks fourteenth in cancer mortality worldwide. Moreover, its incidence and mortality in males increased to the 6th and 9th place among cancers, respectively. Although men are more likely to develop bladder cancer, women often present with more advanced disease and have unfavorable prognosis. This disease can present as non-muscle-invasive bladder cancer (NMIBC), muscle-invasive bladder cancer (MIBC) and a metastatic form of the disease. The overall survival declines dramatically as the cancer progresses, especially when urothelial cells transition from noninvasive to invasive (1, 2). Each stage of the disease has different molecular drivers, and epigenetic dysregulation also plays an important role in the pathogenesis of bladder cancer (3). Furthermore, heterogeneity is a characteristic feature of bladder cancer, which exhibits a wide spectrum of clinical and pathologic features (4). In addition, chemotherapy is an important treatment method for bladder cancer, but chemotherapy has failed in a large proportion of patients with bladder cancer because of the gradual chemoresistance, which leads to the relapse and progression of tumors (5).

Recent advances in the next-generation sequencing technologies have significantly improved our understanding of the genomic landscape and the molecular underpinnings of bladder cancer (6). This review will summarize the molecular mechanism of bladder cancer occurrence, development and metastasis, as well as the sensitivity towards chemotherapy in patients, in order to pave the way for diagnosis, monitoring, prognosis, and personalized care for bladder cancer. Meanwhile, the novel oncogene sequencing method and strategy will also be discussed.

## Bladder Cancer Genomics

Carcinogenesis of cells is a very complicated process (7). At the genomic level, its cancerous mechanisms are mainly involved the following aspects: oncogene activation and overexpression; tumor suppressor gene mutations and deletions; loss of gene repair function; tandem duplication of nucleotide abnormalities in the genome, Microsatellite instability; dysfunction of signaling pathways; cell telomerase overexpression. In 2005, the United States launched the Oncology Genome Research Program (4), and hopes to find out all the oncogene in lung cancer, brain cancer, and ovarian cancer in the next 13 years in order to diagnose and treat these “terminally diseases”. However, each tumor has its own unique genetic blueprint, even the cause of same tumor type is different between individuals. Scientists hope to use the “Tumor Genome Project” to establish global collaboration to unravel the secrets of all cancers and build a shared database. The successful implementation of this program is expected to truly benefit human health.

Bladder cancer genomics is a discipline based on the study of the bladder cancer genome to reveal the mechanism of bladder cancer development. Bladder cancer genomics can be divided into two aspects (8): to identify new mutation sites, genes and corresponding molecular signaling pathways related to bladder cancer, and to analyze the causes of tumorigenesis and development from the gene mutation spectrum.

### Bladder Cancer Genomics

Gene abnormalities can lead to cell cycle disorders, uncontrolled proliferation, and thus tumors. The genomic defects of bladder cancer are complicated, ranging from single DNA mutations to gene polymorphisms to whole or partial deletions of chromosomes.

The study has found that all human malignancies have somatic gene mutations (9). Activation of oncogenes and suppression of tumor suppressor genes are very common. Abnormal activation of oncogenes such as HER2 can facilitate tumor metastasis. Inactivation of *p53* and *p16* and other tumor suppressor genes involved in cell cycle regulation can inhibit apoptosis of tumor cells. Common mutations in bladder cancer include *TP53*, *PIK3CA*, *TSC1*, *FGFR3*, *HRAS*, and *HER2*. Moreover, common abnormal expression genes include *EGFR*, *Ki67*, *PD-L1*, *ERCC1*, and *BRCA1*. Shariat et al. found that the proportion of *p53* and *p16* abnormally expressed in bladder cancer patients were 56% and 54%, respectively. It was highly correlated with muscle-invasive bladder cancer which often indicates a poor prognosis (5). Soria et al. studied the expression of *HER2* in 354 bladder cancer patients and found that *HER2* is highly expressed in more than 32% of patients. Higher expression of *HER2* indicates more aggressive tumor invasiveness (10). Hayashi et al. inhibited highly expressed *HER2* in bladder cancer cell lines and animal models with *HER2* inhibitor T-DM1 and found that T-DM1 has a more prominent inhibitory effect on bladder cancer cell growth than the trastuzumab. In addition, platinum-resistant bladder cancer cells with have higher levels of *HER2* expression. Interestingly, this group of cells is more sensitive to T-DM1, and cells treated with T-DM1 are more prone to apoptosis (11). The above studies indicate that the oncogene *HER2* plays an important role in the pathogenesis of bladder cancer. Inhibition of *HER2* expression and induction of apoptosis can significantly suppress the bladder cancer cell growth. *HER2* mutations are also present in bladder cancer. Tschui et al. studied both exon 19 and exon 20 of *HER2* gene in bladder cancer patients and found that mutations occur in exon 19 (12).

The cancer genome atlas (TCGA) analyzed DNA data from 131 patients with muscle-invasive bladder cancer and identified 32 mutant genes with high frequency. These mutations are mainly occurred in the cell cycle, chromatin regulation and kinase signaling pathways (13). Another comprehensive analysis of the full TCGA cohort of 412 MIBC cases was conducted in 2017. 58 significantly mutated genes (SMGs) were identified in the expanded cohort, in which 34 mutant genes were not identified in previous analysis and 7 mutant genes were identified in more than 10% of samples: *KMT2A* (11%), *SPTAN1* (12%), *ERBB2* (12%), *CREBBP* (12%), *FAT1* (12%), *ATM* (14%) and *KMT2C* (18%). Moreover, 158 genes that were epigenetically silenced were identified by analyzing DNA methylation and gene expression, for example, *SPATC1L* (silenced in 19%), nicotinate phosphoribosyltransferase (*NAPRT*) (13%), Poly(ADP-ribose) polymerase *PARP6* (26%) and latexin (*LXN*) (27%). However, the DNA hypermethylation in the promoter of other tumor suppressor genes were not found, such as *RB1*, *NF2*, *NF1*, *TSC2*, *TSC1*, *PTEN*, and *TP53* (14).

In addition to single DNA abnormality, bladder cancer genomic research also focuses on polymorphism sites. There are multiple gene polymorphisms in bladder cancer such as *ERCC1*, *XRCC1*, *GSTP1*, *CDA*, *GSTM1*, and *GSTT*. Different gene polymorphisms could predict chemotherapy sensitivity in bladder cancer. Xu et al. studied the efficacy of 41 patients at stage IV of muscle-invasive bladder cancer with different *ERCC1* genotypes after 2 to 6 cycles of platinum-based chemotherapy. The results suggest there is a discrepancy of short-term response to chemotherapy and median overall survival in patients with different ERCC1 genotype. Compared with C/T and T/T genotypes, patients with C/C genotype had a better short-term response to chemotherapy and median overall survival (15).

Studies have shown that there are changes in the PI3K, MAPK, Hedgehog, and Wnt pathways in the bladder cancer genome (16–18). Among them, the PI3K pathway is the most studied signaling pathway in bladder cancer. ERBB receptor family is the upstream promoter of the PI3K pathway. For example, ERBB1 (EGFR) can activate the PI3K pathway by activating RAS. Overexpression of EGFR, ERBB2 or ERBB3 is associated with tumor grade, stage, and prognosis in bladder cancer. Moreover, mutations in ERBB2 or ERBB3 have been found in some muscle-invasive bladder cancers (19). *PIK3CA* encodes the catalytic subunit p110α of PI3K, and its mutation occurs in 25% of non-muscle invasive bladder cancer (20). The above studies suggest that the mechanism of bladder cancer development and progression may be related to the PI3K signaling pathway.

### Genetics of Bladder Cancer Cell

Chromosomes are aggregates of genetic material, and chromosomal abnormalities are an important part of the genetic defects of bladder cancer. Based on genetic techniques such as genomic hybridization and loss of heterozygosity, it is found that bladder cancer has complex chromosome number and structural variation. Bartoletti et al. have shown that the partial or complete loss of genetic material at chromosome 9 can lead to the loss of tumor suppressor genes such as *p16* which often indicates the recurrence of low-grade bladder cancer. Abnormal numbers of chromosomes 3, 7, 13, and 17 have also occurred in bladder cancer (21). Among them, chromosome 3 polyploid and chromosome 7 aneuploidy may be related to the progression and malignancy of bladder cancer, and the aneuploidy of chromosome 17 often implies a high recurrence rate of bladder cancer (4, 22).

## Heterogeneity of Bladder Cancer Genome

Tumor heterogeneity describes differences of genotype and phenotype between the same tumor type in different patients, and different sites in an individual, and even between the cancer cells within a tumor. For different individuals, tumor heterogeneity mainly depicts as differences in clinical characteristics such as pathological type, the degree of malignancy, invasion, and metastasis as well as gene mutation, and abnormal expression of proteins. For the same individual, tumor heterogeneity mainly exhibits as differences in gene expression profile and mutation at different sites of the same individual or between the cells of the same tumor, even between many subtypes of tumor cells in the tumor. Phenotypic and genomic (genetic) heterogeneity are two manifestations of tumor heterogeneity. Phenotypic heterogeneity is also the result of genomic heterogeneity to some extent (23). Therefore, the key to the study of tumor heterogeneity lies in genomic heterogeneity research.

According to the biological behavior of bladder cancer, it can be divided into muscle-invasive bladder cancer (MIBC) and non-muscle invasive bladder cancer (NMIBC). Studies have shown that there is a mutation of *Ha-ras/FGFR3* gene in NMIBC, and activation of the mouse *Ha-ras* gene can induce NMIBC, which is rare in MIBC. Inactivation of the *p53*/*Rb*/*PTEN* gene is more likely happen in MIBC. Activation of the *uroplakin II*-specific urothelial-specific promoter in transgenic mice demonstrated the expression of SV40T antigen in the urothelium which can inactivate *p53* and *pRb*, thereby induces the invasion of tumor and metastasis of bladder cancer. Inactivation of *p53*/*Rb*/*PTEN* is less common in non-muscle invasive bladder cancer (24, 25). These findings suggest that the genomic heterogeneity (mutation and expression profile) between MIBC and NMIBC is likely to be significantly different in the biological behavior of the two bladder cancers.

Tumor heterogeneity can also exhibit as heterogeneity at the stage of tumor development. The infinite proliferation of cells is one of the main features of malignant tumors, and this characteristic requires *de novo* synthesis of telomeres by telomerase to prolong the loss of telomere ends in each round of DNA replication. Wu et al. performed whole genome and transcriptome sequencings of 97 bladder cancer patients and found that the telomere reverse transcriptase gene *TERT* is highly expressed in invasive and advanced bladder cancer patients as compared to the early and non-invasive bladder cancer patients. This group of patients has a higher mutation rate of the *TERT* promoter (26). This study showed a significant difference in the *TERT* promoter expression level and the mutation rate between early non-invasive and advanced invasive cancerous cells in bladder cancer.

Bladder cancer tumors have different biological characteristics of the tumor cells subsets, and there are certain differences in gene expression profiles among different cell subpopulations, which is well-described by the heterogeneity within the tumor. The most representative of intratumoral heterogeneity is the presence of a subset of tumor cells with different molecular markers in the bladder cancer. For instance, bladder cancer cells can be divided into common bladder cancer cells and bladder cancer stem cells, with a significant difference in genetic mutations at the latter. Li et al. extracted the single cell genomes of bladder cancer stem cells (BCSCs) and non-bladder cancer stem cells (non-BCSCs) for PCR amplification, and analyzed data from 20 cells. The single cell mutation rate of the *TERT* gene promoter C228T was found to be 50% higher in BCSCs and lower in non-BCSCs (27). There are two main hypotheses of the formation mechanism of malignant tumors heterogeneity in bladder cancer: clonal evolution hypothesis and cancer stem cell hypothesis (28, 29). The clonal evolution hypothesis was proposed by Nowell in 1976 which hypothesize that tumor cells originate from the first generation of single mutant cells. Thus, all tumor cell genomes are identical to the genome of the single cell, but complex factors such as mutations and environmental influences are involved in the proliferation and evolution process. Therefore, the subsequent formation of tumor cells is gradually varied in term of genotypes and phenotypes and eventually result in the tumor heterogeneity. Cancer stem cells are a research hot topic in molecular biology of tumor in recent years. The cancer stem cell hypothesis states that tumor cells are derived from cancer stem cells. Due to the multi-directional differentiation potential of tumor stem cells, various tumor cells proliferate and differentiate to produce subpopulation with different phenotypic functions and thus result in tumor heterogeneity. Kreso et al. found that the mutated genes of tumor cells in the same colorectal cancer case remained unchanged after continuous multiple implantations, but there were significant differences in phenotypes such as proliferative capacity and drug resistance (30). However, this hypothesis does not explain why there are differences in the genome of tumor cells of the same tumor type between different individuals.

Tumor heterogeneity is the result of genetic and environmental interactions. Although the current hypothesis does not completely reveal the cause of tumor heterogeneity, it offers research direction for tumor heterogeneity to some extent.

## Epigenetic and Bladder Cancer Genome

### Telomerase Reverse Transcriptase

Telomerase is a ribonucleoprotein that is essential for the replication of most eukaryotic chromosome ends. In cancer cells, telomerase can be activated. In contrast, telomerase is epigenetically silenced and inactivated in normal cells (31). However, mutations of important genes can reverse telomerase silencings, such as tumor suppressor genes or mutations in the tumor suppressor pathway signaling molecules that may affect telomerase activity in human tumors (32). Telomerase consists of two subunits: telomerase RNA component (TERC) and telomerase reverse transcriptase tert (TERT) catalytic subunit. The *TERT* gene encodes a telomerase reverse transcriptase catalytic subunit and assembles into a ribonucleoprotein protease complex to maintain telomere length which plays an important role in the maintenance of tumor genome stability (33). In recent years, *TERT* promoter mutations have been found in malignant tumors such as melanoma (34) and glioma (35). Studies have shown that the *TERT* promoter mutation is highly correlated with the prognosis of transitional cell carcinoma and confirmed that the −124 C > T mutation (corresponding to C228T) is associated with high expression of *TERT* and telomerase activity (36).

Wu et al. found a coexistence relationship between the *TERT* promoter mutation point and the *TC53*/*RB1* inert somatic mutation point. By determining the chromosomal instability index, it was found that the tumor chromosomal instability index of the *TERT* promoter mutation was significantly higher than that of the tumor without the *TERT* promoter mutation (26). *TP53* is involved in the regulation of telomerase activity, maintenance of gene integrity, inert mutations, and its deletion is associated with chromosomal instability. These studies suggest that the coexistence of the two may have a synergistic effect on the chromosomal instability of bladder cancer (37).

Studies have found that the C228T mutation of the *TERT* promoter often occurs in BCSCs (27). Compared with non-BCSCs, C228T has a significantly higher mutation rate in BCSCs which is consistent with the phenomenon of high C228T expression in stem cells compared to normal somatic cells. Importantly, the *TERT* promoter C228T mutation can convert normal bladder stem cells (NBSCs) into tumor-initiating cells. This mutation is located at the promoter region and can lead to tumors formation which can be considered as tumor promoters. It can be seen that the high mutation rate of *TERT* promoter in BCSCs is a new feature of bladder cancer, but the transformation of NBSCs mutation into BCSCs is complex and further research is needed.

Telomerase activity is observed in almost all human tumor types, thus monitoring the tumor’s telomerase activity will greatly contribute to the diagnosis and screening of bladder cancer. Wu et al. screened for the presence of telomerase reverse transcriptase gene promoter somatic cell mutations by Sanger sequencing in 302 patients with different urological tumors. The result showed that 43% of genitourinary tumors had *TERT* promoter somatic mutations. This has determined high-frequency mutation hotspot of the telomerase reverse transcriptase gene promoter in clinical urogenital (26). In different types of urological organ tumors, the amplitude of somatic mutation of the *TERT* promoter was larger (0-63.7%). The urinary tract cancer has the highest mutation frequency among all, while the prostate cancer showed no mutation.

### Chromatin Remodeling

Chromatin remodeling is an important regulatory mechanism of epigenetics, which is characterized by changes in the nucleosome structure and its relative position to the DNA sequence, and alteration of the accessibility of gene promoter region sequences to further regulate gene expression. Chromatin remodeling works primarily through two pathways: ATP-dependent chromatin remodeling complex, the SWl/SNF complex; histone-modifying enzymes including histone methylation, acetylation, phosphorylation, and ubiquitination (38). Recent studies have found that chromatin remodeling related genes have high-frequency mutations in a variety of tumors including bladder cancer, kidney cancer, gastric cancer, ovarian clear cell carcinoma, breast cancer and glioblastoma (38). Mutations in chromatin remodeling-related genes can cause epigenetic changes in local histone modifications and chromatin conformation in cancer cells, leading to dysregulation of downstream signaling genes, changes in biological behavior such as cell proliferation and apoptosis, leading to tumor development (39).

Gui et al. performed full exon sequencing on 9 patients with transitional cell carcinoma and has found 8 frequently mutated chromatin remodeling genes in 59% of transitional cell bladder cancer: *UTX*, *MLL*-*MLL3*, *CREBBP*-*EP300*, *NCOR1*, *ARID1A* and *CHD6* (40). *UTX* has a number of significantly enriched mutations including 11 nonsense mutations, 4 frameshift mutations, and 1 splice site change. One of these mutations was predicted to be able to truncate the JmjC domain, which is critical for the demethylase activity of the protein product. *ARID1A* has the same mutation pattern as *UTX*. In addition, mutations in the above genes have also been found in other malignant tumors such as ovarian cancer and renal cancer (41, 42). The study found that *STAG2* is located on the X chromosome, encoding sister chromatid cohesion and segregation (SCCS) adhesion complex components, regulating the separation of sister chromosomes during cell division, and inactivation of *STAG2* can cause weakening of chromosome binding and thus aneuploidy (43). Via gene sequencing and rigorous bioinformatics analysis, Guo et al. have found that *STAG2*, *ESPL1* and *NIPBL* genes with frequent mutations in bladder cancer are involved in the SCCS process (44). The study further revealed that patients with mutant *STAG2* had a higher frequency of chromosomal aneuploidy by detecting copy number changes in the chromosome arm, and significantly worse in the prognosis of *STAG2* gene somatic cell mutation in bladder cancer patients compared with *STAG2* bladder cancer patients without the mutation. Collectively, in SCCS, other types of tumors only report rare or low-frequency mutations in oncogenes. Bladder cancer is a tumor type that is first known to have a high-frequency genetic damage gene in the SCCS process in which the genetic mutation rate is approximately 32%. The genetic changes affect the SCCS process which may involve the development of bladder cancer. Despite the detailed mechanism of *STAG2* leading to the pathogenesis of transitional cell carcinoma is still not known, the high-frequency recurrent mutant gene is indeed a new pathway associated with transitional cell carcinoma in the SCCS process of transitional cell bladder cancer (45).

## Oncogene Sequencing Method and Strategy

The traditional sequencing method is Sanger sequencing. However, this method has the disadvantage of low sensitivity and it is difficult to obtain all genomic information, and high cost but low output which limits the application of Sanger sequencing in large-scale sequencing. High-throughput sequencing, the second-generation sequencing technology (also known as next-generation sequencing, deep sequencing) can simultaneously sequence a large number of genes, making it possible to accurately detect tumor and transcriptome abnormalities in tumor patients (46–48). This method can detect tumor genomic abnormalities including nucleotide substitution, insertion and deletion, copy number alteration and chromosome recombination, and improve the detection efficiency and resolution of the genome. As a next-generation sequencing technology, high-throughput sequencing has unparalleled advantages over the traditional sequencing methods. First, high-throughput sequencing can perform large-scale parallel sequencing of a large number of genes, resulting in a remarkable increase in gene detection efficiency. Second, high-throughput sequencing detects the number of occurrences of a certain gene in a sample can reflect the expression level of the gene to some extent. Finally, high-throughput sequencing is more economical than traditional large-scale gene sequencing.

### Whole Genome Sequencing

Whole genome sequencing is the sequencing of the entire genome of tumor tissue with the DNA sequence of the germ cells from the same patient (tumor cell mutation is not present in the germ cells) as a control can effectively identify the changes in all regions including nucleotide replacement, structural rearrangement, and copy number changes (49). Therefore, whole genome sequencing is the most comprehensive depiction of the tumor genome, suitable for the study of tumor genome-wide association. However, whole genome sequencing requires the detection of a large number of sequences is redundant for studies that do not require the whole genome. In this case, shotgun sequencing based on the Sanger sequencing principle is more suitable for identifying somatic cell rearrangements and copy number changes in the genome.

Sanger sequencing is the gold standard for current gene sequencing. Wu et al. performed Sanger sequencing to sequence 302 different urinary tumor patients and found a high-frequency mutation hotspot of *TERT* promoter in urinary tumor patients wherein *TERT* promoter somatic mutation in 55.6% of bladder cancer (26). Guo et al. performed genome-wide sequencing of 99 tumors and corresponding peripheral blood samples from patients with transitional cell carcinoma by Sanger sequencing. SCCS-related gene mutations such as *STAG2* and *ESPL1* were confirmed from 11240 candidate somatic mutations. Moreover, mutant genes such as *FGFR3*, *TP53*, *PIK3CA*, and *RB1* are ubiquitous in malignant tumors (44). Second-generation sequencing invention has received extensive attention at the beginning compared to Sanger sequencing. Andrea et al. recruited 50 patients with muscle-invasive bladder cancer and performed whole-exome sequencing on germline and pretreatment tumor DNA. Then these patients were treated with neoadjuvant cisplatin-based chemotherapy and underwent surgery to evaluate the pathologic response. They identified *ERCC2*, one nucleotide excision repair gene, was only significantly mutated in responders compared with nonresponders (50). David et al. investigated the correlation of *ERCC2* with pathologic response to neoadjuvant cisplatin-based chemotherapy in an independent validation patient cohort, and they confirmed that *ERCC2* was indeed associated with response to chemotherapy (51). Desai et al. used the second-generation sequencing technology to map the mutations of primary and recurrent transitional cell bladder cancer. By comparing the differential expression of tumor-associated DNA damage response genes, they also found that especially *ERCC2* mutations indicate a better prognosis of chemoradiotherapy for transitional cell bladder cancer and a lower rate of recurrence and metastasis within 2 years (52).

### Exon Sequencing

Exon sequencing is a method of capturing whole genome exon DNA by using targeted sequence capture technology to enrich the construction of DNA library before performing high-throughput sequencing. It is characterized by sequencing of the open reading frame without sequencing the introns. Since exons account for only 2% of the entire genome, high-throughput sequencing of the targeted exon greatly reduces the amount of sequencing and achieves higher efficiency. Due to its deep sequencing of the coding region, it can be used to screen for mutations that Sanger sequencing could not. In addition, it is suitable for the study of single nucleotide polymorphism (SNP) and insertion-deletion (InDel) of the target gene. Longo et al. used targeted exon sequencing to detect 50 cases of patients with pTis-pT4b bladder cancer. Through the analysis of staged somatic mutations in patients with such high-risk bladder cancer, the mutated genes and mutation profiles were determined. In terms of single mutations, it was found that epigenetic and cell cycle-regulated genes have significantly higher mutation rates. PI3K/mTOR and cell cycle/DNA repair showed the highest mutation rate among the other assessed pathways. Moreover, *RB1* and *TP53* mutations were found to frequently coexist with *NF1* and *PIK3CA* mutations (53).

However, exon sequencing also has certain limitations. It is unable to sequence non-coding genes and thus fail to conduct a comprehensive study of intron-regulated genes associated with the target gene, and also impossible to identify copies.

### Single Cell Sequencing

In the past, genome sequencing was the extraction of DNA from many cells or a piece of tissue. It is inevitable that sequencing results are obtained from many cell genomes. However, tumors are heterogeneous and the genomic information of different subtypes is more or less different. Investigation of the total DNA information extracted from different cell populations will inevitably cause researchers to ignore the differences between cells to varying degrees and further bias the study of the target cell genome (54, 55). Single cell sequencing is the sequencing of genomes from a single cell. The process involves the isolation of single cells and extraction of DNA followed by single-cell genomic amplification and sequencing analysis. This will prevent the influence of other cell genomes on the sequencing results of the target cells and thus accurately measure the copy number of a single nuclear gene (56).

Bladder cancer is a solid tumor with significant heterogeneity, and the process of research needs to be careful to prevent bias resulted by other cells. In order to study *TERT* promoter mutations in the bladder cancer, Li et al. had isolated the single cells of BCSCs, non-BCSCs, NBBCs, and non-NBBCs and extracted their genomes for single-cell gene amplification and sequencing. The high-frequency mutation sites of C228T were found in the *TERT* promoters of BCSCs and non-BCSCs, and the effect of C228T mutation on bladder cancer and BCSCs was investigated (27). In order to study the genetic basis and origin of bladder cancer stem cells, Yang et al. had performed single-cell sequencing on 59 single cells including bladder cancer stem cells (BCSCs), bladder cancer non-stem cells (BCNSCs), bladder epithelial stem cells (BESCs), and normal bladder epithelial cells. It was found that BCSCs showed clonal homogeneity, and phylogenetic analysis indicated that BCSCs were derived from BESCs or BCNSCs. This study has confirmed that 21 abnormal key genes in BSCSs wherein 6 of which were not reported in bladder cancer in the past, which fully demonstrated the accuracy of single-cell sequencing (57). Faridani et al. demonstrated that microRNAs as the potential markers of different cell types and cell status by performing single-cell sequencing of the small-RNA transcriptome on human embryonic cells and tumor cells (58).

### Transcriptome Sequencing

The transcriptome sequencing is a technique to sequence the transcript RNA, which is a sequence of cDNA that is reverse transcribed from mRNA, total RNA or other RNAs (e.g., small RNAs) to obtain the sum of RNA in a state of the cell. It was found to be sensitive and effective for intron fusion including oncogene activation caused by in-frame fusion. Kekeeva et al. performed high-throughput transcriptome sequencing on bladder cancer patients and screened 4 fusion introns in bladder cancer from 819 suspected fusion introns such as *SEPT9*/*CYHR*, *IGF1R*/*TTC23*, *SYT8*/*TNNI2* and *CASZ1*/*DFFA* (59). Transcriptome sequencing can analyze gene expression profiles to reveal differentially expressed genes in tumors. Based on the transcriptome sequencing data, Zhang et al. confirmed the differential gene expressions of several bladder cancers through the comparison of bladder cancer and normal tissue transcriptome, in which *ELF3* and *MYBL2* are the tumor suppressors while *MEG3*, *APEX1*, and *EZH2* are the inducer of bladder cancer progression (60). In addition, transcriptome sequencing can also be applied to detect somatic mutations, but it is difficult to find a matching normal sample control which limits the application in this aspect. Liu et al. performed transcriptome sequencing of transitional cell carcinoma and identified 937 differentially expressed genes (61).

It is well known that miRNAs can form RNA silencing-inducing complexes to inhibit translation of target mRNAs which is an important mechanism for gene expression regulation. Studies have shown that miRNAs also play an important role in tumor pathogenesis (62). Therefore, transcriptome sequencing technology has broad application prospects in detecting the abnormal miRNA expression in tumors.

## Bladder Cancer Genomics and Precision Treatment

The traditional treatment strategy for bladder cancer is to formulate treatment plans according to grading and staging: early surgical treatment, middle and late stage surgery and perioperative radiotherapy and chemotherapy. However, the efficacy of treatment especially chemotherapy varies greatly in patients with the same type, grade, and stage of histology. It shows that there is still a considerable difference in the real situation of the patients with the same diagnosis. Since the differential sensitivity to chemotherapy in different patients, blind implementation of gold-standard chemotherapy is very likely to delay the treatment of chemotherapy-resistant patients. In response to the above problems, some researchers have proposed a strategy for precision medicine.

The essence of precise treatment of bladder cancer lies in the in-depth explanation of the molecular pathogenesis of bladder cancer. This is demonstrated on a more detailed molecular typing based on genome studies of bladder cancer. In addition, deletions of multiple tumor suppressor genes such as *CDKN2A* and *RB*, and amplification of oncogenes such as *E2F3*, *SOX4*, *EGFR*, and *CCND1* were also identified. By further analyzing the mRNA and protein expression levels of the gene, bladder cancer is classified into 4 types. Type I and type II have the characteristics of breast cancer-like cells namely in which *ERBB2* is highly expressed and estrogen receptor 2 (ESR2) pathway is activated. The difference is that type I mutations with fibroblast growth factor receptor 3 (FGFR3) and papillary histological phenotype III have similar characteristics of basal-like breast cancer cells, with the gene expression profile of squamous cells and stem cells, such as epidermal growth factor receptor (EGFR) and increased expression of keratin 5, 6A and 14 and the like. Type IV is between type II and type III (32). This gene expression-based typing provides a scientific basis for the new molecular typing of bladder cancer. However, the relationship between molecular typing and clinical efficacy and prognosis still needs further exploration.

Genomics research can explore the genes involved in disease recurrence, prognosis, and drug sensitivity to effectively predict and evaluate the efficacy of chemotherapy for bladder cancer. Choi et al. had classified bladder cancer into three types by clustering the gene expression profiles of 183 cases of bladder cancer (6). Among them, luminal- and basal-like cells are same as the type I and type III of the TCGA type. The difference is that bladder cancer caused by luminal-like cells with wild type p53 and its activation of the signaling pathway is divided into a new category, known as the p53-like type. Combined with clinical prognosis and efficacy analysis, the luminal type has the best prognosis and is sensitive to neoadjuvant chemotherapy while basal type has the worst prognosis, about 60% of patients are not sensitive to neoadjuvant chemotherapy. The prognosis of p53-like type is between the two aforementioned, but almost insensitive to neoadjuvant chemotherapy. Choi’s research provides a promising reference for the development of chemotherapy for bladder cancer (63). Roland et al. used a single-sample genomic subtyping classifier (GSC) to predict four consensus subtypes: luminal, luminal-infiltrated, basal, and claudin-low. They validated the clinical impact of these consensus subtypes in independent neoadjuvant chemotherapy (NAC) and non-NAC datasets. Luminal tumors showed the best overall survival (OS) with and without NAC. Luminal-infiltrated tumors were associated with poor prognosis with and without NAC. Compared with surgery alone, basal tumors had the most improved OS with NAC. Claudin-low tumors had poor OS regardless of treatment regimen (64). Despite the current lacking of well-defined predictive genetic markers in bladder cancer, above studies provide a clear research direction for precise treatment.

In addition, bladder cancer genomics can reveal the molecular mechanisms involved in the development and progression of bladder cancer and provide new molecular targets for the treatment of bladder cancer. To date, targeted therapy has achieved outstanding results in a variety of cancer treatments, such as gefitinib targeted therapy in treating lung cancer. However, bladder cancer targeted therapy is still at the early stage of clinical research, thus targeted therapy is not the option in guideline treatment recommendations for bladder cancer. At the present, some high-frequency abnormalities of bladder cancer that have been discovered and identified by genomics may become potential therapeutic targets. This includes signaling pathways such as EGFR, P13K/mTOR, HER-2 and FGFR3. Notably, the Food and Drug Administration recently approved erdafitinib as a treatment for patients with advanced urothelial cancer (UC) with FGFR3/2 mutations, who progressed on platinum-based chemotherapy. Clinical trials showed the overall response rate of erdafitinib was 49% in patients with *FGFR3* mutations (65, 66). Secondly, it is immunotherapy such as PD-1, and CTLA4. PD-1/PD-L1 immunotherapy can enhance the body’s immune response to kill tumors and is effective for a variety of tumors including bladder cancer, lung cancer, stomach cancer, and kidney cancer. The US Food and Drug Administration (FDA) has approved this therapy for bladder cancer. Interestingly, bladder cancer has many tumor mutation burden (TMB). By analyzing the 443 bladder cancer samples from TCGA, single nucleotide polymorphism (SNP) and C>T were the most frequent mutation types, and significant differences in tumor immune microenvironment were observed between the low TMB group and the high TMB group, including Mast cells resting, NK cells resting, T cells CD4 memory activated and T cells CD8, which makes it sensitive to immunotherapy (67). However, some patients is resistant to PD-1/PD-L1 immunotherapy, partially because TGF-β in fibroblasts reduces anti-tumor immunity by inhibiting CD8+ T cell infiltration into the tumor parenchyma (68).Fortunately, a personalized neoantigen-based vaccine, NEO-PV-01, was designed by whole exome and RNA sequencing of each patient’s formalin-fixed tumor and matched normal cells from blood, and it consisted of high-quality neoepitopes encoded by somatic mutations and selected using bioinformatics algorithms. The clinical trial showed NEO-PV-01 in combination with PD-1 blockade was a safe and effective treatment to patients with bladder cancer by inducing T cells to traffic to the tumor and mediate cell killing (69). The third is cell cycle regulatory molecules, such as Aurora kinase A and Polo-like kinase I (3). The fourth is the antibody-drug conjugate (ADC), for example, enfortumab vedotin comprises an anti nectin-4 monoclonal antibody, protease cleavable linker and monomethyl auristatin E (MMAE). Because nectin-4 is highly expressed in all metastatic UC tumors, once the bonded enfortumab vedotin is internalized, the microtubule-disrupting agent MMAE is released leading to apoptosis of the tumor cell. Clinical trials demonstrated enfortumab vedotin is a new therapeutic method in patients with platinum- and immune checkpoint inhibitor-refractory disease (70, 71).

## Conclusion and Prospect

As shown **Table 1**, many genes are involved bladder cancer progression and metastasis, such as *FGFR3*, *TP53*, *EGFR*, *HRAS* and *Ki67* (72–74), as well as *HER2*, *TSC1*, and *ERCC1* which are the genes associated with bladder cancer. Among them, *HER2* could be the potential therapeutic target, and *ERCC1* is involved in the drug resistance of cisplatin and other chemotherapeutic drugs. Low expression of *ERCC1* has a longer survival period. *BRCA1* has DNA repair function and reduces the effect of chemotherapy drugs (12, 75, 76). However, the current molecular markers that predict the prognosis and chemotherapy efficacy of bladder cancer patients are still at premature stages. Genetic markers that can accurately predict the therapeutic effect of bladder cancer is yet to found. In the future, intensive studies are needed to define the precisive molecular characterization of bladder cancer (77). Genomics is an effective tool for studying the molecular pathology of bladder cancer. It is believed that with advances in sequencing technology, genomics may bring more help to the targeted therapy of bladder cancer (78).

**Table 1:** Common gene alterations in bladder cancer.

Gene Name	Function	Reference
*HER2*	Higher expression of *HER2* indicates more aggressive tumor invasiveness.	(10)
*ERCC1*	*ERCC1* is involved in the drug resistance of cisplatin and other chemotherapeutic drugs. Low expression of *ERCC1* has a longer survival period.	(13)
*EGFR,ERBB2,ERBB3*	Overexpression of *EGFR, ERBB2* or *ERBB3* is associated with tumor grade, stage, and prognosis in bladder cancer.	(19)
*PIK3CA*	Its mutation occurs in 25% of non-muscle invasive bladder cancer, associated with bladder cancer development and progression.	(20)
*p16*	The loss of tumor suppressor genes (*p16*) often indicates the recurrence of low-grade bladder cancer.	(21)
*p53,Rb,PTEN*	Inactivate *p53* and *pRb* induces the invasion of tumor and metastasis of bladder cancer.	(24, 25)
*TERT*	*TERT* is highly expressed in invasive and advanced bladder cancer patients as compared to the early and non-invasive bladder cancer patients.	(26)
*STAG2*, *ESPL1*, *NIPBL*	*STAG2*, *ESPL1* and *NIPBL* genes with frequent mutations in bladder cancer are involved in the sister chromatid cohesion and segregation process.	(44)
*ERCC2*	*ERCC2* mutations indicate a better prognosis of chemotherapy for bladder cancer and a lower rate of recurrence and metastasis within 2 years.	(50–52)
*ELF3*, *MYBL2*	Tumor suppressors.	(60)
*MEG3*, *APEX1*, *EZH2*	Inducer of bladder cancer progression.	(60)
*FGFR3*	The luminal-papillary subtype is characterized by *FGFR3* mutations, fusion with transforming acid coiled-coil containing protein 3 (*TACC3*), or amplification.	(65)

## Author Contributions

JZ and CL designed and supervised the review. YL and LS searched literature and wrote the manuscript. XG and NM reviewed of the manuscript. All authors contributed to the article and approved the submitted version.

## Funding

The authors disclosed receipt of the following financial support for the research, authorship, and/or publication of this article: This work was supported by the National Natural Science Foundation of China (81672956, 81972390, 82002566), Beijing Science and Technology Projects (Z181100003818003), Beijing-Tianjin-Hebei Basic Research Cooperation Special Project (19JCZDJC65800(Z)), China Children and Teenagers’ Fund (Y1803001), and Key Clinical Projects of Peking University Third Hospital (BYSY2017001).

## Conflict of Interest

CL was employed by Beijing Zhongke Jianlan Biotechnology Co., Ltd.

The remaining authors declare that the research was conducted in the absence of any commercial or financial relationships that could be construed as a potential conflict of interest.
